# The Dual Character of Animal-Centred Care: Relational Approaches in Veterinary and Animal Sanctuary Work

**DOI:** 10.3390/vetsci12080696

**Published:** 2025-07-25

**Authors:** Anna K. E. Schneider, Marc J. Bubeck

**Affiliations:** 1 FAU Kompetenzzentrum für interdisziplinäre Wissenschaftsreflexion, Friedrich-Alexander University Erlangen-Nürnberg, 91054 Erlangen, Germany; anna.schneider@fau.de; 2 Faculty for Health Sciences Brandenburg, University of Potsdam, 14469 Potsdam, Germany

**Keywords:** animal rights, animal welfare, care ethics, euthanasia, qualitative methods, working with animals

## Abstract

Caring for the lives and welfare of animals is central to veterinary and animal sanctuary work, yet the meaning remains a subject of complex debates. Different stakeholders negotiate what constitutes appropriate care, leading to conflicting demands and expectations from internal and external sources. This article is based on two qualitative studies: Study I explores the multifaceted aspects of death work in farm animal medicine, emphasising the practical, emotional and ethical challenges involved. Study II examines human–animal interaction in sanctuaries, which reveal tensions between instrumental and relational care in animal-centred work. Relational care represents a subjectifying approach with individual attention to animals, while instrumental care is a more objectifying perspective based on species representation. These demands can often be contradictory, complicating day-to-day decision making under pressure. To analyse these complexities, this study employs Clarke’s situational analysis (social worlds/arenas mapping), providing a means of comparing care work across different fields. This approach highlights how actor constellations, institutional settings, and structural constraints influence the negotiation of care. Addressing these issues provides a more nuanced understanding of the professional challenges of animal-centred care and the necessary skills to navigate its inherent contradictions.

## Simple Summary

Caring for animals is central to veterinary and animal sanctuary work. However, the meaning of animal-centred care is often the subject of debate. This article explores the challenges faced by professionals when balancing two approaches: treating animals as individuals and considering their species-specific needs. These tensions are further shaped by the differing expectations of various groups—including animal owners, caretakers, veterinarians, institutions, and the public—who may hold conflicting views on what constitutes appropriate care. This article focuses on human–animal interactions in sanctuaries and the difficult decisions regarding killing an animal in veterinary medicine, such as euthanasia. The two care approaches and the expectations of actor groups can conflict—particularly when professionals must make decisions in real-life situations that are constrained by limited resources and time. By highlighting these tensions, this study provides insight into the nuances of animal-centered care and the skills needed by professionals to navigate the ever-increasing complexities of the field.

## 1. Introduction

Caring for the lives and well-being of animals is central to the professions of veterinary and animal-sanctuary work. In both fields, the meaning of animal-centred care is the subject of complex debates. For example, what are an appropriate space and structure for tigers in captivity or how to decide whether to treat or kill an animal in veterinary care. Different actors negotiate what constitutes appropriate care for animals in different ways. As a result, people who work professionally with animals face conflicting demands and expectations. These can come from within, from organisations and professional groups themselves, or from outside, from politics, the media or social movements. In addition to conflicting expectations, people working with animals have to make welfare (The term ‘animal welfare’ is understood in this text as the endeavour of ensuring the well-being of animals; it considers the facts that the term is viewed as scientifically controversial, interpreted in different ways, and critically discussed) decisions every day, often under less-than-ideal conditions, such as time pressure or limited financial or human resources [1,2]. These decisions reflect different forms of care: instrumental care, which focuses on ensuring species-appropriate standards and organisational efficiency, and relational care, which is shaped by empathy, trust, and close interaction with individual animals. What is considered appropriate care is not only negotiated among actors but also shaped by broader cultural and historical contexts. The meaning of good care is always embedded in specific social constructions of human–animal relationships, which shift over time and across societies [3,4,5]. These dimensions should therefore be an integral part of how we understand professional animal work.

Care work with animals is closely linked to questions of animal ethics. The discussion centres on safeguarding the welfare of animals. This can be viewed both from the perspective of *animal rights* and from the perspective of *animal welfare*. Animal welfare refers to an individual’s state in coping with its environment, encompassing physical health, emotional states, and adaptive capacities [6]. Welfare assessments are based on measurable conditions and not merely on whether living circumstances are ‘natural’. This broader scientific understanding provides an important framework for evaluating animal-centred care practices. While animal rights emphasise the subjective rights of animals, animal welfare advocates for better, more species-appropriate animal husbandry. In terms of content, there are parallels here with the care work explained above. The American Veterinary Medical Association defines animal welfare as the humane responsibility for the well-being of animals, including housing, management, disease prevention, care, and (if necessary) euthanasia [7]. This definition reflects a primarily instrumental understanding of care, where structured provisions are made for animals’ physical needs. In contrast, ethical approaches, such as that of Palmer and Sandøe [8], emphasise the promotion of individual autonomy and development, aligning with what can be described as a relational understanding of care. These perspectives are also reflected in Nussbaum’s capability approach, which argues for a dignified life for nonhuman animals that goes beyond basic welfare and includes emotional and developmental well-being [9].

When considering care work for animals and the associated practices, the understanding of animal welfare must be addressed. Institutional and individual interpretations influence the ways in which animals are cared for and in which care work is considered necessary. Care ethics offer a theoretical approach to the context-sensitive analysis of professional human–animal relationships [10]. Care encompasses a broad spectrum of behavioural practices that can also have negative effects [11]. For both professional groups, these practices are crucial to their understanding of their roles and shape their interactions.

In addition to the individual actors involved in animal-centred care work, the institutional setting must also be considered closely. The influence of institutions begins with the categorisation of animals based on laws or internal guidelines. Animals categorised as companion animals generally benefit from stronger protections than those classified as livestock. This disparity prompts ethical concerns about how different species are valued and the consequences for care practices. For example, farm animals are often treated with less regard than pets, sparking discussions about the moral implications of these distinctions [12]. These classifications influence the rights and protection of animals in everyday life and determine which actions are considered permissible, particularly in care work. In most cases, veterinary clinics and animal welfare organisations set standards that regulate interactions with animals and define treatment options.

Furthermore, institutional norms and cultures characterise everyday working life and determine the availability of time and financial resources. For example, tight schedules and limited resources in veterinary clinics increase pressure on staff, while financial constraints in animal welfare organisations limit the capacity to care for animals. Hierarchies and role allocations also have an influence on work with animals.

In this article, we explore the tensions inherent in professions that work with and care for animals. In doing so, the multiple stakeholders and expectations that shape animal-centred care are taken as our starting point. The argument is based on two major empirical studies: Study I examines the multi-dimensional aspects of killing in farm animal medicine, highlighting the ethical, emotional and practical demands placed on practitioners [13]. Study II focuses on human–animal interactions in different contexts, particularly in sanctuaries, with a particular emphasis on the challenges and complex negotiations inherent in caring [14]. These empirical perspectives allow us to trace how instrumental and relational care are enacted, justified, and negotiated in two highly contrasting institutional settings. To analyse these tensions, we draw on Clarke’s [15] situational analysis and the social worlds/arenas theory developed by Strauss [16]. This theoretical framework offers tools for analysing how groups interact within specific social domains. Social worlds are dynamic, permeable groupings of actors connected through shared activities and identities, continuously shaped by processes of interaction and negotiation. Social arenas are understood as focused spaces of negotiation in which different social worlds (conflictually) exchange views on topics to assert their interpretations and, in some cases, assert them against others. The theory is characterised by the multi-perspectivity of social situations. This approach allows us to conceptualise animal-centred care as a negotiated practice shaped by overlapping actor constellations, institutional logics, and material conditions.

Through the comparative analysis, we demonstrate how the various expectations of stakeholders, including caretakers, veterinarians, animal owners, and government bodies, give rise to a complex arena in which animal-centred care work is negotiated. Building on this, we analyse how these external demands intersect with the dual approach of care in practice: instrumental care, which is oriented towards species-level objectivity, and relational care, which emphasises individualised, empathic engagement. This article provides an in-depth understanding of professional animal-centred care work and the complex negotiations involved, demonstrating how diverse stakeholder expectations intersect with the dual approaches of care, thereby intensifying the ethical and practical dilemmas faced by those in animal-related professions.

## 2. Materials and Methods

The present article employs a comparative approach to explore the complexities of animal-centred care work across two institutional contexts: sanctuaries and veterinary medicine. Utilising a qualitative research methodology, the care dynamics, responsibilities and ethical dilemmas that practitioners encounter in these two settings are analysed. The comparison allows not only for the specific challenges within each context to be illuminated but also for a more extensive understanding of how stakeholder expectations, institutional structures, and resource constraints influence care practices and interactions.

To achieve this objective, a range of qualitative research methods was employed, including semi-structured interviews and ethnographic observations, with a view to gathering rich, nuanced data. These methodologies facilitated the unearthing of the intricacies inherent in care work, encompassing the frequently disregarded emotional burdens and moral considerations that practitioners are required to navigate. In the context of care work, practitioners are continually interpreting the actions, needs and circumstances of other actors. Given the plurality of actors involved in care work—each bringing their own situational definitions and knowledge frameworks—an ecological approach is adopted to analyse these “*ecologies of knowledge*” [17]. This perspective emphasises the relationships and interdependencies within animal-centred care work, exploring how different stakeholders contribute to and negotiate the meaning of care [15].

To illustrate these dynamics, we draw on two extensive empirical studies: Study I examines the multifaceted aspects of killing in veterinary medicine, focusing on how veterinarians navigate the boundary work of determining what constitutes a ‘good’ or appropriate death. This research spans various practices, motives, and fields within veterinary medicine, such as small and large animal care. Study II explores human–animal interactions in sanctuaries, emphasising the complex relational dynamics that arise in these settings. Collectively, these studies enhance our comprehension of the multifaceted actors involved in care work and the intricate interplay between instrumental and relational care. Following the presentation of these individual study designs, the comparative approach is introduced (for an overview, see the COREQ checklist [18] in the Supplementary Materials).

### 2.1. Study Design I: Veterinary Medicine

In the field of veterinary medicine, daily activities encompass the care and treatment of animals, in addition to their killing [19,20]. This empirical study examines the intertwining of death work and veterinary professionalism, with a particular focus on the co-constitution of professional identity and ethical boundary making within veterinary practices [13]. This study adopts a grounded theory (GT) approach [21], exploring these complexities inductively with a view to contributing to the relatively underexplored field of sociological research on veterinary work [22]. Given the relative paucity of comparative sociological research on veterinary practices, this study is exploratory in nature [23]. The challenges associated with smaller sample sizes in comparative designs are mitigated by grounded theory principles such as constant comparison and theoretical sampling, which prioritise depth and theoretical relevance over representativeness.

#### 2.1.1. Data Collection

The data collection process was conducted in accordance with the principles of constructivist grounded theory [21]. To capture both minimal and maximal contrasts within the sample, 17 veterinarians with diverse specialisations and disciplines were interviewed. The participants represent a broad spectrum of practice areas (e.g., small animal medicine, large animal medicine, exotic species), years of experience, specialties, gender, ages, geographic regions, and socioeconomic backgrounds.

Semi-structured interviews were conducted between 2020 and 2022 and lasted between 45 min and 2 h [24,25]. The interviews explored topics such as daily routines, killing practices, ethical dilemmas, and the professional biographies of veterinarians (see the Supplementary Materials). To complement the interviews, relevant documents such as guidelines, manuals, and websites were analysed to capture the discursive and material dimensions of killing practices within veterinary medicine.

#### 2.1.2. Data Analysis

The analysis combined Charmaz’s [21] constructivist grounded theory methodology with Clarke’s [15] situational analysis (SA) to ensure a nuanced and comprehensive interpretation of the data. MAXQDA 24 software was utilised for systematic coding, thereby facilitating the identification of emergent themes and patterns. In the first stage, inductive open coding was applied to the interview transcripts. These were read multiple times in order to identify meaningful units within words, phrases, and sentences. These units were then given conceptual labels (codes) that reflected the participants’ professional experiences. Similar codes were then grouped into broader conceptual categories. The initial coding process was guided by an awareness of the specific field of veterinary work. In the second phase, focused coding was employed to summarise and refine the material by identifying the most significant and recurring categories. This enabled a more abstract understanding of the professional logic of care to be achieved. Finally, theoretical coding integrated the findings with concepts within interdisciplinary debates in the sociology of professions, human–animal studies, and veterinary ethics. The study employed SA mapping strategies, including situational, social worlds/arenas, and positional maps, to visually represent the diverse and complex field of veterinary death work [26].

The establishment of interdisciplinary interpretation groups was a key aspect of the study, with the objective being to enhance the rigour of the analysis. The focus of these groups was on the validation of coding, as well as mapping and the refinement of emerging theoretical insights. This collaborative process enhanced intersubjectivity and ensured a thorough interpretation of the data.

#### 2.1.3. Research Reflexivity and Ethics

The dual positionality of the researcher, who is both a veterinarian and a sociologist, provided unique access and profound field insights. This unique vantage point enabled an in-depth comprehension of the multifaceted professional and ethical complexities inherent in killing practices. However, this positionality also gave rise to challenges related to potential biases. To address these challenges, the researcher engaged in continuous reflexivity, documenting reflections throughout the research process and aligning the study with the interpretive goals of sociological inquiry.

Ethical approval was obtained from the Research Ethics Committee of the Faculty of Social Sciences, LMU Munich (GZ 23-13). All participants provided written informed consent, ensuring ethical standards were upheld.

### 2.2. Study Design II: Rescue Centre

This study examines care work in animal sanctuaries, specifically focusing on a ‘big cat’ sanctuary, to explore the dynamics of human–animal interactions and the roles of various stakeholders in care practices. Sanctuaries provide a unique setting for understanding how caregiving is shaped by both institutional structures and the interpersonal relationships between humans and animals. The ethnographic field study, conducted between 2019 and 2020, included visits on public open days and during non-public hours to observe caretakers’ routines. Interviews combined spontaneous conversations on open days with scheduled appointments outside public hours. The study included interviews with six randomly selected volunteers to explore their backgrounds and motivations. At the time, two permanent caretakers were employed, and a third, in the hiring process, was unexpectedly available for an interview. Informal conversations with a visiting veterinarian and a former intern further supplemented the data.

#### 2.2.1. Ethnographic Study

The ethnographic interviews, integrated with observations, were semi-structured to encourage more natural dialogue [27]. As rapport developed between the researcher and participants, this flexible format maintained the authenticity of the interactions. This approach is in line with Mayring’s [28] recommendation to adapt methods to the research subject rather than rigidly applying pre-defined structures. Volunteers and carers were actively engaged in tasks, and interviews often took place alongside their regular duties, sometimes interrupted by responsibilities. Informal conversations were beneficial, allowing interviews to emerge naturally from ongoing interactions. The interview guide remained flexible, with questions evolving in response to the flow of the conversation.

Participant observation complemented the interviews by providing deeper insight into animal-centred care practices. According to Atteslander [29], participant observation involves “*systematically capturing, recording, and interpreting perceptible behavior at the time of its occurrence*” (p. 73). A key feature of this method is the researcher’s personal involvement in the participants’ interactions. As Mayring [28] explains, researchers become part of the social system they observe. Participation varied from full involvement in work tasks to observing caregivers’ routines [30]. The researcher’s role remained transparent, and participants were aware that the observations complemented the interview data. Observations were conducted across four different contexts and with varying levels of active participation: (1) open days, (2) active participation in a work assignment, (3) accompanying caretakers in their daily routines, and (4) observing caretakers and the veterinarian during vaccinations. These observations were not standardised but were guided by a clear focus on human–animal interactions and care relationships [31].

#### 2.2.2. Data Analysis

Data analysis is based on a comprehensive dataset of semi-structured interviews, ethnographic field notes, visual materials (including photographs), and reflective memos. This approach provides a rich understanding of participants’ experiences and their context. Interviews were audio-recorded, transcribed, and thematically analysed using MAXQDA 12 software. Initial coding followed a grounded theory approach, revealing key themes that reflect the complexity of human–animal care work in the sanctuary. Ethnographic field notes added context, highlighting social interactions and care practices, and were analysed using the same thematic framework to identify patterns consistent with the interview data. Thick descriptions captured the emotional and relational dynamics of care work, while visual material was analysed alongside verbal data to deepen the understanding of participants’ experiences. Reflective memos documented the researcher’s evolving thoughts and biases, enriching the analysis by reflecting on the dynamics between participants and the challenges of care work.

Building on these findings, social worlds/arenas mapping was employed to visualise the relationships between different actors and arenas in the sanctuary setting. This method helped create a map representing the interconnected social worlds and the dynamics that shape animal-centred care work. The map synthesises both instrumental and relational aspects of care work, offering a clear, spatial representation of how different roles and structures interact. The synthesis of these strategies provides a nuanced understanding of the interplay between instrumental and relational care work in the sanctuary, highlighting the importance of context and interpersonal relationships in shaping care practices.

### 2.3. Comparative Approach Using Social Worlds/Arenas Mapping

Social worlds/arenas theory provides a robust framework for investigating the complexities of professional practices involving animals within institutional settings. By emphasising the interactions and negotiations between diverse actors, this approach allows for a detailed exploration of how multiple stakeholder perspectives, institutional norms, and material conditions shape professional care practices.

To operationalise this framework, Clarke’s [15] situational analysis (SA) was employed as a methodological tool. SA integrates the principles of grounded theory with mapping strategies, thereby enabling the systematic visualisation and analysis of relations within complex situations. Specifically, social worlds/arenas maps were constructed based on empirical data from the two case studies. These maps focus on the primary arena of animal-centred care work, with the various social worlds involved surrounding it. The maps represent relationships and attributes such as boundary permeability (e.g., dashed lines for porous boundaries) and size, with the latter indicating empirical prominence.

The subsequent synthesis of the data was facilitated by Clarke’s mapping strategies, resulting in the creation of separate maps for each case study to identify key actors, relationships, and institutional structures. These maps were then used as analytical tools to explore how stakeholder expectations and institutional norms shape care practices.

The comparative approach was designed to synthesise findings from the two case studies, focusing on shared challenges and institutional distinctions. The maps enabled the identification of overlapping social worlds and arenas, allowing for the abstraction of broader dynamics that define animal-centred care work. Through this process, we explored how the duality of care—instrumental and relational—is shaped and intensified by external and internal demands across these professional contexts.

The integration of qualitative methods with situational analysis (social worlds/arenas mapping) enabled the study to transcend mere descriptive analysis, thereby providing a conceptual framework for comprehending the tensions and ethical dilemmas that are intrinsic to animal-centred care work. This methodological integration provides a rigorous basis for addressing the central aim of the paper, which is to illuminate how institutional structures and stakeholder negotiations co-construct the dual responsibilities of care in animal-oriented professions.

## 3. Study I: The Dual Approaches of Care in Veterinary Work

In veterinary medicine, caring for animals involves not only healing but also killing, which has an ethically ambivalent role. Veterinarians often perceive killing as a form of care, particularly when it is used for the purpose of alleviating suffering or for ethically justified purposes [20,32,33]. Veterinary care operates within a dynamic interplay of societal human–animal relationships, institutional frameworks and professional interactions [34,35,36]. A complex interplay shapes how care is understood, legitimized, and provided. Today, veterinary medicine is a specialized profession with various practices, settings, and roles. Farm animal medicine differs greatly from companion animal medicine because farm animals are considered not only patients but also production units [37]. These social categorizations influence the goals of veterinary care, shifting the focus from the health and well-being of patients to functionality and economic prosperity. This ambivalence is reflected in—and influences—the negotiation of relational and instrumental approaches to care.

### 3.1. Contextualizing Care in Farm Animal Medicine: Legal and Social Ambivalences

The ambiguity of the animal is reflected in its legal status in Germany. According to the German Civil Code (§90a BGB), animals have a special legal status that is distinct from property but not quite that of a legal subject. This tension between object and subject status exemplifies the ambivalent social perception of animals. Further distinctions, such as between livestock and companion animals, illustrate the legal nuances in veterinary practice. Horses, for example, occupy a unique position within the law and veterinary care [38]. Depending on their classification as food-producing animals, there are restrictions on the use of medicines that have a significant impact on their medical treatment. This classification highlights how the legal and institutional framework influences care practices and the ethical challenges faced by veterinarians.

In addition to legal issues, veterinary care is further complicated by the expectations of various stakeholders. These include media portrayals, the demands of animal welfare organisations, and the advocacy of law reform movements. A vet working with farm animals spoke about the challenges of balancing the demands of different parties. She talked about the negative portrayal of farm animal medicine in the media, as well as legal restrictions and the focus on profitability in this field: “*We’ve got a bit of a bad reputation, to be honest. There was a really nasty article in DIE ZEIT that called us ‘drug dealers in green coats’, which is just not true. On the one hand, we’ve got major restrictions on the active ingredients we’re allowed to use for farm animals, and on the other, we’ve got to meet strict diagnostic requirements. The more we do, the more difficult it is to balance being economical and legal*.” (veterinarian interview). Each stakeholder brings different and sometimes conflicting priorities to the table, adding to the complex landscape in which veterinarians operate.

Veterinary care is further complicated by its triadic structure, involving the veterinarian, the animal, and the client. Ethical tensions arise when the interests of these parties diverge, particularly in matters of prioritisation [39,40]. The role of the veterinarian is often debated: Is their function akin to that of a mechanic, whose duty is to follow the client’s instructions, or closer to that of a paediatrician, who advocates for the best interests of the dependent patient?

Law [41] extends this framework by suggesting two additional *“objects of care”* that influence veterinarians: the wider societal responsibilities (‘the bigger picture’) and the veterinarians themselves. These levels illustrate how the demands on veterinarians vary from case to case, shaped by the complex web of actors, institutions, and societal expectations.

All of the interviewed vets emphasised that their work is always about the animal: “*I like (...) working with animals, I think they’re just very, very pleasant creatures*” (veterinarian interview). Despite this, safe food production was another central motive, as exemplified by the statement of one vet: “*I like being around people and animals. And working in food production makes sense to me, it’s something relevant and important to us as a society*” (veterinarian interview). Veterinary work is not only imbued with the social values of caring for animals but also of producing safe food: “Even this work in agriculture, I just see as something that is relevant, something that is important for us as a society” (veterinarian interview). This balancing of priorities often places veterinarians in ethically complex situations where food safety and economic considerations influence care decisions, particularly in relation to euthanasia.

### 3.2. Balancing Instrumental and Relational Care

In the context of farm animal medicine, instrumental care refers to interventions aimed at preserving animal health and functionality within a production system, which often prioritizes productivity, biosecurity, and herd management. In contrast, relational care focuses on the well-being of individual animals and the emotional and ethical connections among veterinarians, animals, and owners.

A farm veterinarian provided a detailed account of the treatment of a cow suffering from milk fever, a condition that required immediate action to save the cow’s life and preserve her productivity. The veterinarian’s intervention was guided by expert knowledge of the disease’s pathophysiology and the practical goal of restoring the animal’s productivity. While undoubtedly lifesaving, this care primarily reflects an instrumental approach in which the cow’s value is closely tied to her function within the production cycle. Relational aspects, such as the veterinarian’s emotional connection to the animal or the animal’s individual experience, remain implicit or subordinated. This points to structural constraints that discourage deeper individual engagement. This is illustrated by the description of a female veterinarian, in which the physiological state of the animal is in the foreground and the act of killing is portrayed as a relief: “*For me, it wasn’t too bad because the animal was only released. I saw the whole story and it was hopeless, and the animal had suffered a lot. It was a trapped cow, and for me, it was really about simply releasing the animal from its suffering*” (veterinarian interview). However, the interviews also include examples in which animals are euthanised because appropriate care is not possible on the farm. In such so-called “*animal welfare cases*” (veterinarian interview), veterinarians must make decisions that reveal the limitations of both instrumental and relational care. These cases are perceived as particularly distressing. These cases take an emotional toll on veterinarians, reflecting their ethical ambivalence and tension. Deelen et al. [42] highlighted the incongruity between ethical principles, such as the ‘best interest’ of the animal, and the pragmatic realities that veterinarians encounter in their daily practice.

Another veterinarian described a situation where she treated a farmer’s “*favourite cow*” (veterinarian interview). Despite her best efforts, the cow’s health deteriorated due to complications from a claw resection, and the cow was ultimately sent to the slaughterhouse, a decision the veterinarian later regretted. She expressed her remorse, stating, “*In hindsight, I wish we had euthanised her earlier. The journey to the slaughterhouse could have been spared; it was not necessary*” (veterinarian interview). This case exemplifies the conflicting demands of care: the veterinarian’s emotional engagement with the individual animal and the institutional pressures that prioritise the cow’s classification as a productive resource. The veterinarian’s reflections underscore the tension between relational care, grounded in empathy for the animal, and the structural realities of farm animal medicine, which often limit such considerations. The “*favourite cow*” case is an example of a rare moment when relational care takes centre stage. The veterinarian treats the animal not only as a patient or production unit but also as an individual with whom the farmer and the vet share a history. The subsequent regret about not euthanizing the animal earlier suggests dissonance between the veterinarian’s emotional commitment and the institutional requirement to preserve economic value for as long as possible. These moments of moral distress reveal how relational care persists in the background and is often in tension with dominant expectations.

These cases demonstrate the intertwining of instrumental and relational care in farm animal medicine. Although care is typically provided within a framework that emphasizes productivity and efficiency, veterinarians also engage in emotionally charged, ethically complex practices that demonstrate their commitment to the well-being of individual animals. As one veterinarian insightfully observed, “*We’re in a bind—our clients are the animal owners, but we’re also responsible for the animals’ welfare*” (veterinarian interview). The persistence of relational care, even under institutional constraints, reveals its enduring role in farm veterinary medicine. Recognizing this dual approach of care can improve our understanding of the emotional labour and moral tensions that shape everyday veterinary practice.

## 4. Study II: The Complex World of Care Work in Rescue Centres

While veterinary medicine highlights tensions between relational and instrumental care in a clinical setting, animal sanctuaries offer a different institutional and emotional landscape. Here, the data reveal complex dynamics between these two forms of care, shaped by nuanced human–animal interactions, as well as structural challenges. Caregivers operate within a framework shaped by institutional constraints, resource limitations, and the individual needs of the animals. These challenges reveal how the practical and emotional aspects of animal-centred care intersect, affecting both the carers and the animals.

### 4.1. Care Work in the Sanctuary: Context and Challenges

“*The most important task as an animal carer? That my animals are well. Quite simply, that my animals are doing well*” (caregiver interview).

The selected wildcat sanctuary operates with a mix of full-time staff, volunteers, and interns who care for animals from a variety of backgrounds, including zoos, circuses, and private ownership. Although the organisation sees itself as a temporary sanctuary, ideally with the goal of finding better permanent homes, many of the hard-to-rehome wild cats end up staying there permanently. New staff at the sanctuary, often trained in hierarchical zoo environments, undergo a transition to its more personalised yet administratively complex working structure, where unclear leadership, differences in expertise among team members, and chronic understaffing pose significant challenges. As one of the few full-time staff members summarised it, “*I feel more responsible here than at the zoo*” (caregiver interview). However, this increased responsibility comes with considerable challenges, as professional staff with specialist knowledge must work with volunteers who may lack comparable knowledge yet are actively involved in decision-making processes. This interplay reveals that relational care, grounded in responsibility and emotional commitment, may be disrupted by institutional or knowledge-based hierarchies—an often-overlooked aspect of animal-centred work. This dynamic often causes conflict or inefficiency due to mismatched expertise and authority within the sanctuary team. The sanctuary’s outdated enclosures and financial constraints compound the challenges. While efforts to improve living conditions continue, these constraints affect both the welfare of the animals and the daily routines of their caretakers.

### 4.2. Settling In: Individualised Care and Challenges

The individualised care of animals at the sanctuary begins with their admission and acclimatisation. An example of this process is illustrated by an employee’s account of a serval: “*With him [pointing to Serval] it’s just a difficult story, because he came from this flat, that is, he was used to people, this woman also told us that he sometimes slept in bed with us [sic], [...] but when he was here, he didn’t show himself to be accessible to us at all, so really zero*” (caregiver interview). In cases like this, a longer familiarisation process ensues, wherein staff members work to build trust through food and play. However, disruptive events—such as necessary veterinary check-ups or vaccinations—can hinder this progress, creating a delicate balance between granting the animal freedom and familiarising it with new living conditions. As one staff member notes, “*Well, when an animal is new here, you actually try to leave it as undisturbed as possible at the beginning, so that it can acclimatise in peace, i.e., to build up as little pressure and stress as possible, but on the other hand I at least do it in such a way that I show myself to the animal a lot, even during normal procedures, so that the animal also gets to know it, so don’t wrap it in cotton wool, it should get to know the noises and everything that goes on here every day*” (caregiver interview).

This acclimatisation process is further complicated by the inherent dangers of the animals, which limits direct access for handling. For example, moving animals from outdoor to indoor enclosures is a necessary task due to legal housing requirements, cleaning schedules, or significant temperature drops. Additionally, some animals respond differently to individual carers or volunteers. Employee R describes a female cougar as a “*cat for women*” and notes that she is “*female-oriented*” (caregiver interview). This observation prompts a male colleague to invest extra effort in building rapport with her: “*I realised that the puma is afraid of me because she doesn’t know me at all. She’s mistrustful of men anyway, but she gets on really well with [staff member R] and not with me. So, I spend more time with the puma now, bribing her with a little milk [...] to show her that I’m not so bad*” (caregiver interview). Such relational care fosters trust, which is essential for the daily interactions between caregivers and the animals.

### 4.3. Instrumental Care: Practical Responsibilities

Instrumental care encompasses the practical, routine tasks required to maintain animal welfare, such as enclosure maintenance and health interventions. These practices are primarily guided by species-specific standards and legal requirements, often carried out under considerable time and resource constraints. Daily cleaning routines are complemented by periodic deep cleaning and the replacement of bedding, requiring considerable effort and volunteer involvement. For instance, staff must move logs, branches, and other materials to ensure the animals’ environments remain enriching. While some changes provide enrichment, others can be challenging for individual animals, particularly those with specific needs, such as the caracal that is blind in one eye. To accommodate its spatial awareness difficulties, the staff has opted to maintain a stable habitat to allow the animal to navigate comfortably.

The sanctuary adheres to the principles of behavioural enrichment, incorporating various structures and toys to enhance the animals’ daily lives. For example, after Christmas, staff collect discarded Christmas trees, whose unique scents and textures provide stimulating experiences. One caretaker reflects, “*So we make an effort, I’d like to do a bit more, but there’s often not enough time to really sit down and prepare and make something more elaborate, which is why it’s usually something that you just put in or hang up, which doesn’t require much effort*” (caregiver interview). Due to limited resources, care that extends beyond basic provisioning often relies on external support, such as workshops with visitors and volunteers to create enrichment elements from materials like fire hoses or car tires.

Health care is another crucial aspect of instrumental care. The sanctuary collaborates with a local veterinarian who has experience with exotic animals. Routine veterinary visits, including vaccinations, require meticulous planning and preparation. For large cats like tigers and pumas, vaccinations can occur from a distance using a blowgun, while smaller animals necessitate direct injections. Staff utilise specially designed boxes to facilitate safe and close contact during these procedures, although such preparations require time and are often constrained by daily schedules and safety regulations.

Despite the necessity of veterinary interventions, these visits are often negative experiences for both animals and staff, compounded by the inability to communicate the underlying intentions of care: “*They don’t realise that we’re only doing them good, for them the negative experience, the prick, the stress, that just stays there*” (caregiver interview). This communicative barrier highlights a fundamental tension within relational care: while caregivers may act out of genuine concern, their inability to explain or justify these actions to the animals undermines the trust they seek to build. The resulting mismatch between intention and reception is not only emotionally taxing but also ethically charged, as it confronts the limits of empathy and understanding across species boundaries. This dimension becomes particularly relevant when comparing professional care in sanctuaries with veterinary contexts, where emotional bonds and practical interventions are continuously re-negotiated. Efficiency and pragmatic implementation take precedence, often forcing caregivers into ethically and emotionally challenging interactions; for example, caregivers may use sliding doors as lures to encourage animals closer to the blowgun. Staff often feel a palpable sense of resentment toward the stressful impact of these procedures on the already tense animals, and each successful vaccination is met with relief—not only for procedural success but in order to preserve a fragile relational dynamic.

### 4.4. Relational Care: Building Trust

In contrast, relational care emphasises the establishment of trust and understanding between the caregivers and the animals. Daily interactions are crucial for rebuilding and maintaining this trust, even amid intrusive medical measures. “*I had picked up a pretzel with butter from the bakery earlier and was eating it when I broke off a piece to give to her. Surprisingly, she was completely relaxed, even though we had just caught her half an hour earlier using significant restraint, and I had to hold her tightly to prevent her from biting. Despite that, she sat calmly in front of me looking out and taking the pretzel from me. I found it fascinating. People often say that we don’t want to harm them, and perhaps she sensed that. Yet, the very act of capturing her with force could have made her want to withdraw completely. I found that really impressive*” (caregiver interview). This moment encapsulates a fundamental tension within caregiving practices: the caregiver’s emotional investment and ethical intent may not always coincide with species-specific welfare protocols. The act of offering a buttered pretzel—perhaps motivated by a desire to comfort or reestablish connection—underscores the practical and ethical complexities of navigating affective responsiveness within the constraints of institutional care. Such seemingly minor gestures illuminate broader ambiguities inherent in sanctuary-based caregiving, where anthropomorphic inclinations, shifting care standards, and affective bonds frequently intersect. Rather than interpreting these actions solely as errors or lapses in judgment, we propose that they be understood as illustrative of the ongoing negotiation that defines care work—an arena marked by the continual recalibration of ethical ideals, material constraints, and the lived dynamics of interspecies relations. Caregivers recognise the downsides of their work, grappling with the potential consequences for their relationships with the animals, particularly concerning health-related interventions that may involve difficult decisions, such as euthanasia.

In the sanctuary, interactions between humans and animals are shaped by a physical division of their living spaces, highlighting themes of proximity and distance. Unlike pets that are integrated into human households, wild animals reside within human structures that are tailored to meet their needs. Consequently, these encounters occur in a liminal space that considers the animals’ natural requirements alongside human factors, including architectural and financial constraints and practical caregiving issues. Following the social worlds/arenas theory, it becomes clear how many different social worlds interact in the realisation of such measures. Within the animal sanctuary, the carers’ groups coordinate their activities with volunteers and association boards. At the same time, the worlds of visitors, institutions, and regulating authorities are also affected. The desire to improve husbandry conditions is complex and requires the purchase of land, which affects financing and utilisation requirements. Food donations also fluctuate in quantity and quality, influenced by various external actors, such as slaughterhouses and hunters.

In summary, the analysis of care work in the sanctuary reveals a nuanced interplay between instrumental and relational care. While instrumental care encompasses the practical aspects of animal welfare, relational care highlights the importance of building trust and understanding between carers and animals. Sanctuary staff strive to navigate these dimensions while meeting legal and public expectations, ultimately aiming to provide a safe and nurturing environment for the animals in their care. Sanctuary staff treat each animal as an individual from the moment they arrive, taking into account their specific needs and personality traits. Their training enables them to professionally assess and interpret animal behaviour, fostering an intimate understanding of each creature. However, this intimacy is balanced by the need for professional distance, especially during veterinary visits or rehoming. Caregivers must sometimes prioritise the wider interests of animal welfare over personal emotional relationships. Animal welfare legislation imposes further restrictions on care practices, requiring a balance between legal requirements and the needs of the animals. Public pressure for transparency and efficiency often leads to conflict and funding challenges. Therefore, effective sanctuary care requires recognition of these complexities and the development of strategies that balance financial support, legal frameworks, public perception, and internal communication.

## 5. Negotiating Dual Approaches of Animal-Centred Care Work

This comparative study explores the inherent tensions within professions dedicated to animal care. Based on two empirical cases from veterinary medicine and animal sanctuaries in Germany, we analysed how animal-centred care is shaped by negotiations between stakeholder expectations, institutional frameworks, and professional judgement. These care practices occur within a broader context in which various logics of action—economic, legal, relational, and ethical—intersect.

Our findings confirm two aspects of care: instrumental care, which is focused on standardised and efficient procedures that are appropriate for the species in question, and relational care, which is characterised by empathy, individual attention, and trust. Rather than existing as distinct approaches, these forms of care occur together in everyday practice. Professionals and caregivers must constantly adapt to situational demands and ethical complexities by navigating between them.

**Figure 1:**
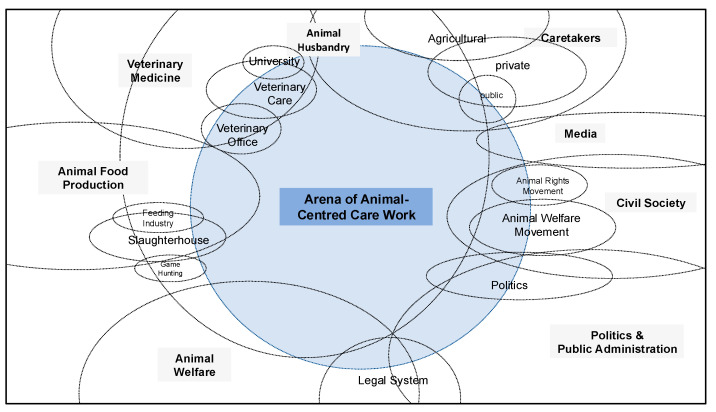
Arena of animal-centred care work.

To visualise this complexity, we created a social worlds/arenas map (see Figure 1), which illustrates the variety of groups involved in animal-centred care work. Rather than aiming for completeness, the map emphasizes key tensions to facilitate comparative analysis of animal-centred care work. The central blue area represents the dynamic space in which care is provided. The surrounding, intersecting social worlds demonstrate how diverse institutions, professions, and movements influence the meanings and boundaries of care. The open worlds on the margins reflect the openness and fluidity of social processes, where boundaries are permeable and roles are continually evolving.

Veterinary medicine is a prominent feature on the left, encompassing practices, clinics, and offices that are responsible for medical treatment, public health, and legal enforcement. This sector is closely intertwined with the fields of animal food production, including the feeding industry, slaughterhouses, and game hunting, where economic and legal demands dominate. Below this cluster, animal welfare emerges as a scientific and legal domain, bridging research, ethical discourse, and regulatory policy.

In contrast, the right side of the map is shaped by civil society actors, particularly animal welfare and animal rights movements. These groups exert influence on public discourse, shape legislation, and often challenge institutional practices. This sphere intersects with the media, which reflects and shapes societal attitudes towards animals. Also depicted are caretakers, whose roles span private homes, public institutions, and sanctuaries, each with different care principles and constraints. Finally, political and public administration fields connect and mediate between legal, scientific, and public demands.

This visualisation highlights a key point: care work is not carried out in isolation but is embedded in a web of competing expectations and value systems. The complexity of this arena necessitates a flexible and responsive approach to care that incorporates structural and relational considerations. For instance, veterinary professionals must comply with regulations and manage efficiency while responding empathetically to animal suffering. Similarly, sanctuary staff must balance intimate, trust-based relationships with practical constraints and safety rules.

Both study settings reveal an ongoing interplay between instrumental and relational care, which is influenced by institutional frameworks, societal expectations, and situational demands. Care is not purely objective or entirely empathetic but, rather, a dynamic practice that is negotiated within a given context. In farm animal medicine, instrumental care often dominates due to species-level objectives such as productivity, food safety, and cost efficiency [37]. However, this does not exclude relational elements: veterinarians report emotional moments such as regret over euthanasia decisions or recognition of animals as sentient beings [13,41]. These moments demonstrate that relational care can emerge even within structured, efficiency-driven systems. In rescue centres, relational care is more explicit [43]: caregivers build trust and form individual bonds with the animals in their care. Nevertheless, instrumental concerns remain central. Practical demands such as limited resources, safety protocols, and legal compliance necessitate a structured approach to care [44]. Even trust-building processes, such as acclimatisation, are influenced by institutional constraints. This interplay between institutional mandates and ethical imperatives often generates complex dilemmas, particularly in cases where an animal’s well-being conflicts with prescribed protocols [45]. Together, these cases demonstrate that animal-centred care involves an ongoing negotiation between ethical sensitivity and pragmatic demands, with professionals continually adapting their approach.

While this study focuses on institutional and professional negotiations surrounding animal-centred care, it is important to acknowledge a further dimension: the role of animals as more than passive recipients of care. Although not the primary focus here, emerging research in animal studies and multispecies ethnography emphasises the significance of animal agency in shaping care relations. Moments of trust, resistance, or behavioural expression on the part of animals are not merely background conditions but can actively influence how care is enacted and experienced. Recognising this dimension further underscores the complexity of care as a negotiated and relational practice [11,41,46].

Therefore, care is always situated. It reflects the professional, cultural, and institutional environment in which it occurs. In the German context, for instance, this encompasses robust legal frameworks for animal welfare, relatively high levels of public awareness of animal rights, and substantial civil society involvement. While these findings may not be directly generalisable to other socio-legal contexts, they offer important insights into how care practices are embedded in broader societal frameworks. Ultimately, we argue that adequate, animal-centred care requires a continual negotiation between the instrumental and relational dimensions. Neither dimension is sufficient on its own. Only by acknowledging this duality can professionals and caregivers respond ethically and effectively to the complex and often contradictory demands of their work.

## 6. Conclusions

In light of these findings, the following conclusion summarises the key insights and implications for professional care work. Animal-centred care work in veterinary medicine and animal sanctuaries is characterised by two approaches: while instrumental care focuses on species-based needs, relational care emphasises the individual and relational aspects. Those working with animals are faced with the challenge of balancing ethical responsibilities with economic constraints and institutional conditions. Structural frameworks play a central role in shaping care practices by categorising animals, defining regulatory guidelines, and determining available resources. These structures reflect societal values and can either constrain or enable the quality of care. The valuation of the two care approaches is negotiated through a social process involving different actor groups. Addressing the dual demands of care requires not only policy reforms to ensure adequate resources, but also a cultural shift that recognises care as a core professional and ethical practice.

## Data Availability

The anonymised data used to support the conclusions of this article can be provided by the authors.

## Supplementary Materials

The following supporting information can be downloaded at: https://www.mdpi.com/article/10.3390/vetsci12080696/s1, Overview of Both Study Designs; Interview Guide for Study I: Veterinary Medicine.
